# A systematic review and meta-analysis of follicle-stimulating hormone levels among men with type 2 diabetes

**DOI:** 10.1186/s12610-025-00257-2

**Published:** 2025-03-14

**Authors:** Fahimeh Ramezani Tehrani, Vida Ghasemi, Marzieh Saei Ghare Naz

**Affiliations:** 1 Reproductive Endocrinology Research Center, Research Institute for Endocrine Sciences, Shahid Beheshti University of Medical Sciences, Tehran, Iran; 2 Department of Midwifery, Asadabad School of Medical Sciences, Asadabad, Iran; 3 Foundation for Research & Education Excellence, Vestavia Hills, Alabama, USA

**Keywords:** Meta-analysis, Follicle stimulating hormone (FSH), Type 2 Diabetes (T2DM), Metabolic, Gonadotropins, Impaired Glucose, Méta-analyse, Hormone folliculostimulante (FSH), Diabète de Type 2 (DT2), Métabolique, Gonadotrophines, Glycémie altérée

## Abstract

**Background:**

There are some studies with inconsistent results regarding the association between follicle stimulating hormone (FSH) levels and type 2 diabetes (T2DM) among men. We performed a systematic review and meta-analysis that explored the FSH levels among men with and without T2DM.

**Results:**

Twenty studies with a total sample size of 4,208 (2167diabetic men and 2041 control) were included in this meta-analysis. The standardized mean differences (SMD) in men who had T2DM compared to control group were -0,237 (CI95%: -0,582 to 0,108; *P* = 0.17; I^2^: 95,83%; Egger's test: 0.06; Begg's test: 0.15). This finding was significant after sensitivity analysis. Among Asian studies SDM was -0,955 (CI95%: -1,630 to -0,279; *p* = 0.006; I2: 96.91%; Egger's test: 0.03; Begg's test: 0.01), with diabetic men had lower FSH than control group. African diabetic males the FSH levels was not different than non-diabetics (SMD: 0,386; CI95%: -0,0401 to 0,813; *p* = 0.07; I2: 94.26%; Egger's test: 0.31; Begg's test: 0.21). Also, among European men the FSH levels was significantly different than non-diabetics (SMD: 0,273; CI95%:0,0960 to 0,450; *p* = 0.003; I2: 18.41%; Egger's test: *P* < 0,0001; Begg's test: 0.31).

**Conclusion:**

Our meta-analysis of the current literature suggests that serum FSH levels are significantly lower in Asian men diagnosed with T2DM compared to their non-diabetic counterparts. This finding highlights a potential association between altered FSH concentrations and the pathogenesis of T2DM. Future studies should aim to unravel these mechanistic pathways and to assess the clinical utility of FSH as a biomarker for T2DM risk assessment and management in the male population.

**Supplementary Information:**

The online version contains supplementary material available at 10.1186/s12610-025-00257-2.

## Résumé

**Contexte:**

Des résultats incohérents concernant l’association entre les taux d’hormone folliculo-stimulante (FSH) et le diabète de type 2 (DT2) chez les hommes ont été rapportés entres différentes  études. Nous avons réalisé une revue systématique et une méta-analyse pour explorer les niveaux de FSH chez les hommes avec et sans DT2.

**Résultats:**

Vingt études portant sur un échantillon total de 4208 personnes (2167 hommes diabétiques et 2041 témoins) ont été incluses dans cette méta-analyse. Les différences moyennes standardisées (DMS) chez les hommes atteints de DT2 par rapport au groupe témoin étaient de -0,237 (IC95 % : -0,582 à 0,108 ; P = 0,17 ; I2 : 95,83 % ; Test d’Egger : 0,06 ; Test de Begg : 0,15). Cette constatation était significative après analyse de sensibilité.

Parmi les études asiatiques, la DMS était de -0,955 (IC95 % : -1 630 à -0,279 ; p = 0,006 ; I2 : 96,91 %; Test d’Egger : 0,03 ; Test de Begg : 0,01), chez les hommes diabétiques qui avaient une FSH plus basse que le groupe témoin. Les niveaux de FSH chez les hommes diabétiques africains n’étaient pas différents de ceux des non-diabétiques (DMS : 0,386 ; IC95 % : -0,0401 à 0,813 ; p = 0,07 ; I2 : 94,26 %; Test d’Egger : 0,31 ; Test de Begg : 0,21). De plus, chez les hommes européens, les niveaux de FSH étaient significativement différents de ceux des non-diabétiques (DMS : 0,273 ; IC95 % : 0,0960 à 0,450 ; p = 0,003 ; I2 : 18,41 %; Test d’Egger : P < 0,0001 ; Test de Begg : 0,31).

**Conclusion:**

Cette méta-analyse de la littérature actuelle suggère que les taux sériques de FSH sont significativement plus faibles chez les hommes asiatiques diagnostiqués DT2 par rapport à leurs homologues non diabétiques. Cette découverte met en évidence une association potentielle entre les concentrations modifiées de FSH et la pathogenèse du DT2. Les études futures devraient viser à démêler ces voies mécanistes, et à évaluer l’utilité clinique de la FSH en tant que biomarqueur pour l’évaluation et la gestion du risque de DT2 dans la population masculine.

## Introduction

Type 2 diabetes mellitus (T2DM) is one of the most common public health issues worldwide, causing harm to the lives of men and women [1]. It is projected that the rising trend of diabetes will grow from 537 million in 2021, to 783 million by 2045 [2]. Diabetes is more prevalent among males than females [3]. Men with diabetes are vulnerable to different cardiovascular and non-cardiovascular complications which can contribute to sexual dysfunction, including erectile dysfunction [4].

The main risk factors for diabetes included lifestyle factors, medical condition factors (obesity, hypertension, cardiovascular disease), hereditary factors (ethnicity and family history), psychological factors, aging and male gender [5]. Endogenous hormone disturbances are other factors that contribute to development of diabetes [6].

Recently, the potential association of T2DM and endogenous hormones like gonadotropins are increasingly followed with interest. There were a number of studies to investigate the associations of follicle stimulating hormone (FSH) with T2DM among males. Some studies reported that men with T2DM had lower levels of FSH compared to the control group [7–10]. Another study found that men with T2DM had higher levels of FSH than the control group [11]. While others failed to support any significant differences between FSH levels among diabetic and non-diabetic men [12–14]. The literature requires to be reviewed comprehensively. FSH as a trophic hormone, mainly acts on the gonads of men, but its extra-gonadal function in bones, adipose tissue, cardiovascular system and immune systems is also being revealed [15]. FSH secrete as a result of a complex interplay among the testis and hypothalamus/pituitary glands [16]. Men with elevated FSH are susceptible to abnormality in semen parameters [17]. Interestingly, FSH Therapy has been administrated for improving reproductive ability of men [18, 19]. FSH is also involved in cardiovascular related functions like protein synthesis, metabolism, angiogenesis, cell division, differentiation and growth [20]. Additionally, abnormality in FSH levels have been associated with cardio-metabolic outcomes and impairment of inflammatory and immune response [21].

There is a lack of systematic reviews and meta-analysis on the existent evidence concerning the possible association between FSH levels and T2DM among males. Given the diverse body of studies, we conducted this review to shed light on the associations between FSH levels and T2DM among males.

## Methods

We followed the guidelines for Preferred Reporting Items for Systemic Reviews and Meta-Analysis statement [22].

Also, this systematic review and meta-analysis was registered with The International Prospective Register of Systematic Reviews (PROSPERO) (CRD42025634103).

### Records identification

Electronic searches of studies published from inception to until January 2025 were performed on PubMed, Scopus, Web of sciences, Cochrane Library, Epistemonikos. There was a restriction on English language and no restriction on publication year. The search was performed using the relevant keywords. Supplementary Table 1 shows the search strategy.

To identify additional studies, references in the included studies were hand-searched. The selection process of articles was performed on the Endnote (version X8, Thomson Reuters, New York, NY).

**Fig. 1:**
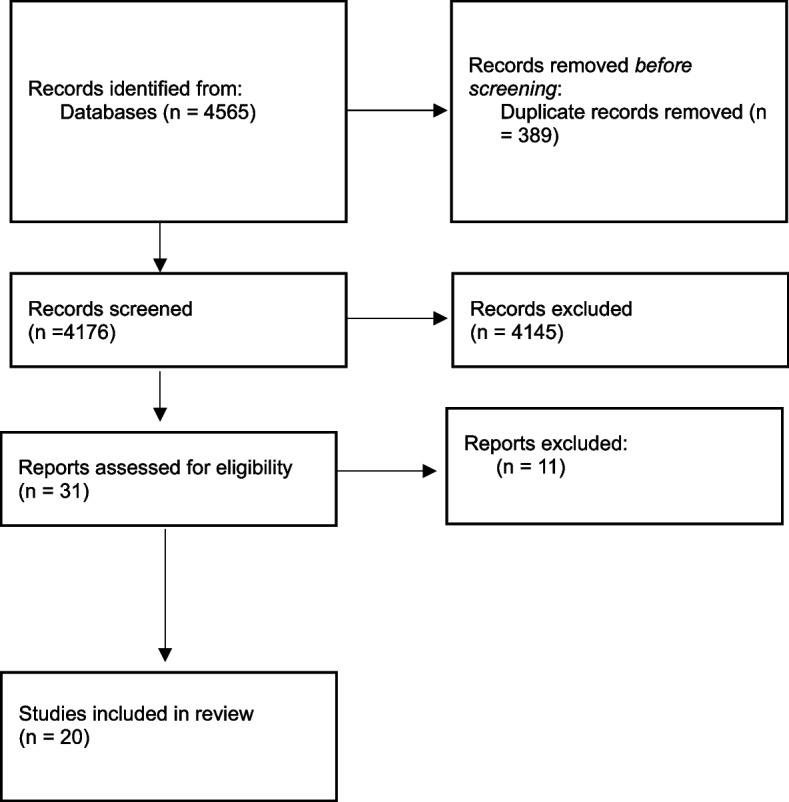
Flowchart of included studies

This process is presented in Fig. 1.

### Study selection criteria

The eligibility criteria for this study were as follows:

- Patients (P): Men with T2DM; Exposure (E): FSH measurements; Control (C): individuals without T2DM; Study outcomes (O): T2DM; Study design (S): observational studies or baseline data of interventional studies.

The exclusion criteria were as follows: lack of controls; studies with insufficient data, abstracts, reviews, commentaries.

First, inclusion criteria were evaluated through the titles and abstracts of the studies identified in search. Then the full texts of the potentially eligible articles were assessed.

### Data extraction

Data extraction was performed by the two reviewers (VGH and MSG). The extraction of data including (author, publication year, country, sample size, mean, SD) was performed by SPSS (version 22, Microsoft Corporation, Redmond, WA). Any disagreements were resolved by discussion with a third person (FRT).

### Quality assessment

The quality assessment of included studies was explored using the Newcastle–Ottawa Quality Assessment Scale (NOS) for observational studies was used for [23, 24]. According to the NOS, scoring was based on the selection of subjects, comparability of study groups, and the assessment of exposure. Discrepancies were resolved by team discussion. The total score was rated from 0 to 9 points (low quality: 0–3, moderate quality: 4–6, and high quality 7–9). Any discrepancies were solved by consensus.

## Statistics

All data were pooled as standardized mean difference (SMD) with 95% confidence interval (CI). Estimated effects and 95% confidence interval (CI) of all the included studies was summarized in a forest plot. The heterogeneity was assessed using the I-squared (I^2^) statistic. Random-effect models were applied in cases with significant heterogeneity or *I*^*2*^ was > 50%. The Egger's test and Begg's test were applied to evaluate publication bias. Subgroup analyses were performed to investigate the potential heterogeneity in different continents. MedCalc® Statistical Software version 22.009 was used to conduct the meta-analysis.

## Results

### Search results

A total of 4565 related publications were obtained from the five electronic databases. After removing 389 duplications, the titles and abstracts of the remaining articles were assessed, and 31 were selected for full-text analysis (Fig. 1).

Eleven studies were excluded due to reasons including reporting effect size for prediabetes or diabetes (combine participants), ineligible data, and unclear type of diabetes [7, 14, 25–33].

Finally, 20 studies that met the eligibility criteria were included in the review (sample size men with diabetes = 2167, sample size control group = 2041).

Among included studies 10/20 were performed in Asia, 8/20 in Africa and 2/20 in Europe. The characteristics of the studies included are shown in supplementary Tables 2.

Among all studies, 7 studies reported that diabetic men had higher levels of FSH [11, 34–39], 6 studies reported lower levels of FSH among diabetic men than control [8–10, 40–42], 7 of them found non-significant differences FSH [12, 43–48].

All studies had moderate to low risk of bias. The supplementary Fig. 1 shows the quality assessment results of included studies.

### FSH levels among diabetic and non-diabetic men

**Fig. 2:**
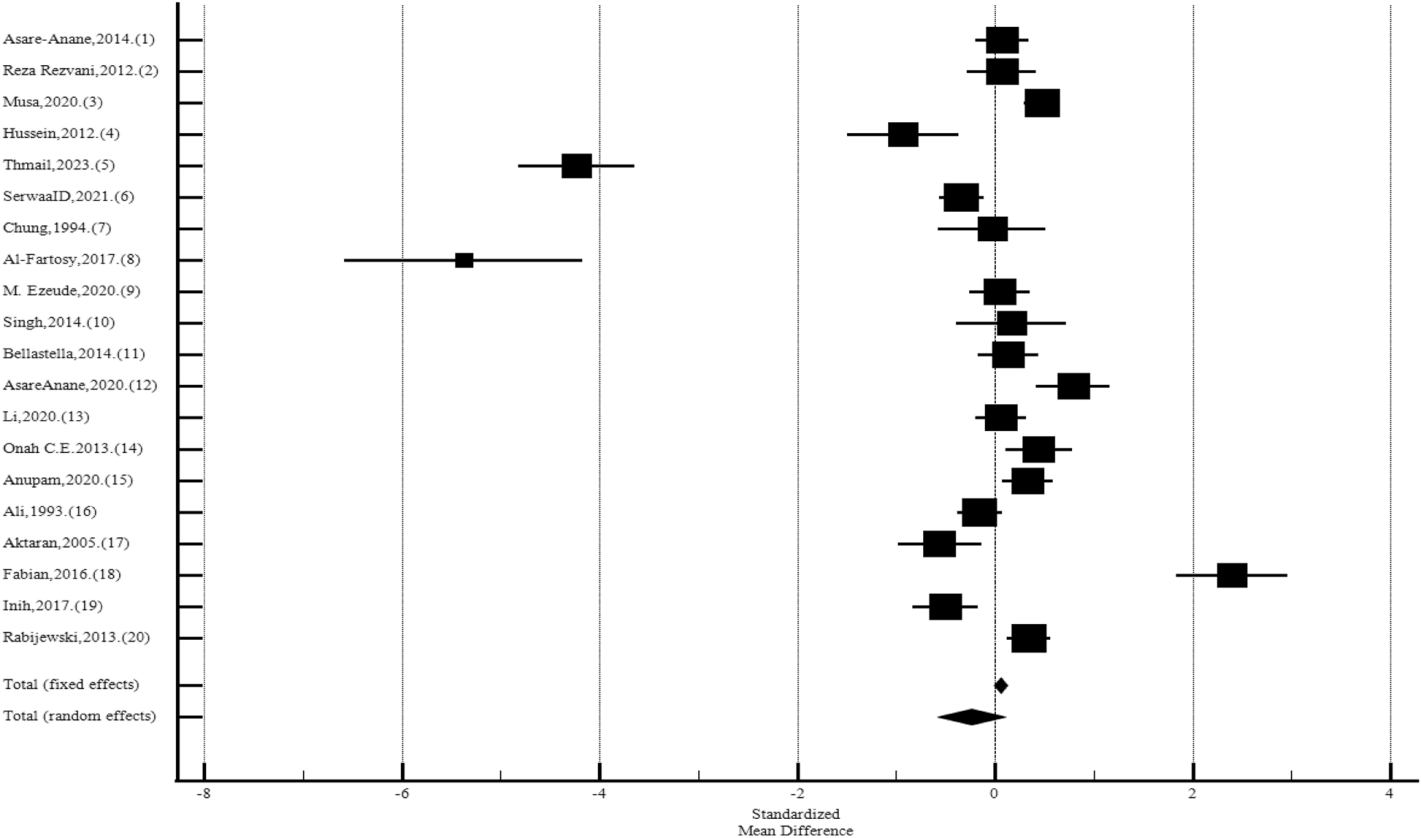
Forest plots of meta-analysis of the levels of Follicle-Stimulating Hormone among the diabetic and non-diabetic ones. Data are pooled SMDs with 95% CIs

Twenty studies reported the mean (SD) of FSH between men with and without diabetes. The pooled standard mean difference of FSH was −0,237 (CI95%:–0,582 to 0,108; *P* = 0.17; I^2^: 95,83%; N_diabetic_ = 2167, N_control_ = 2041; Egger's test: 0.06; Begg's test: 0.15), with diabetic men having lower levels of FSH than those without diabetes (Fig. 2).

**Fig. 3:**
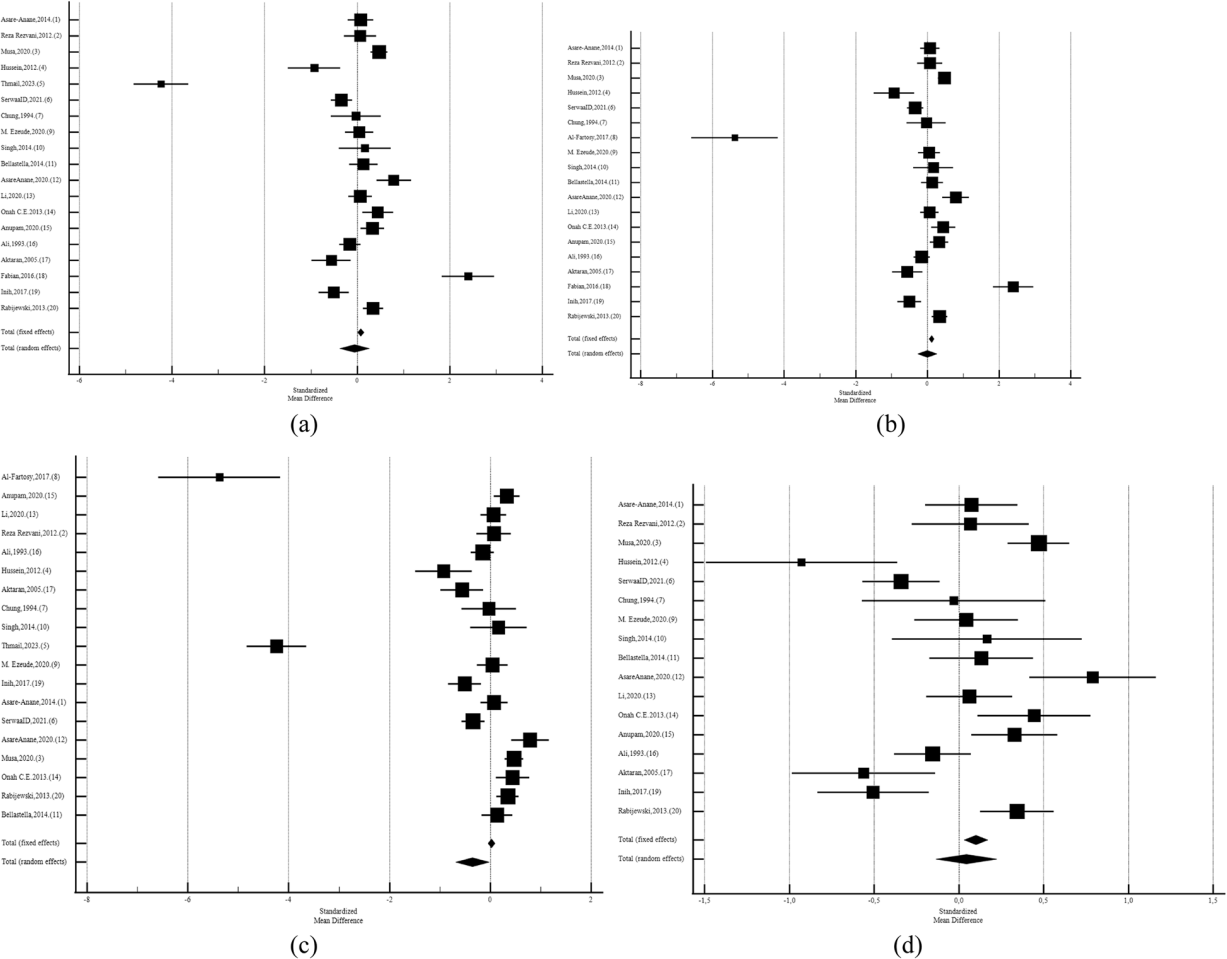
Forest plots of meta-analysis of the levels of Follicle-Stimulating Hormone among the diabetic and non-diabetic ones. Data are pooled SMDs with 95% CIs. **a** after leave out Al-Fartosy,2017. (8), **b** after leave out Thmail,2023. (5) **c**) after leave out Fabian,2016. (18) **d**) after leave out three studies

After leaving out studies that performed by Thmail,et al. (2023) [9] (SMD: −0,002;CI95%:−0,268 to 0,262;*P* = 0.98;I2: 92,17%; Egger's test:0.23; Begg's test:0.46),Al-Fartosy,et al. (2017) [42] (SMD: −0,0575;CI95%:−0,377 to 0,262;*P* = 0.72;I2: 95,18%; Egger's test:0.26; Begg's test:0.34), Fabian,2016. (18) (SMD: −0,356;CI95%:−0,685 to −0,0264;P = 0.03;I2: 95,34%; Egger's test:0.005; Begg's test:0.02), and after leave out three of them at the same time (SMD: 0,0446;CI95%:−0,134 to 0,223;*P* = 0.62;I2: 95,34%; Egger's test:0.005; Begg's test:0.02) (Fig. 3).

**Fig. 4:**
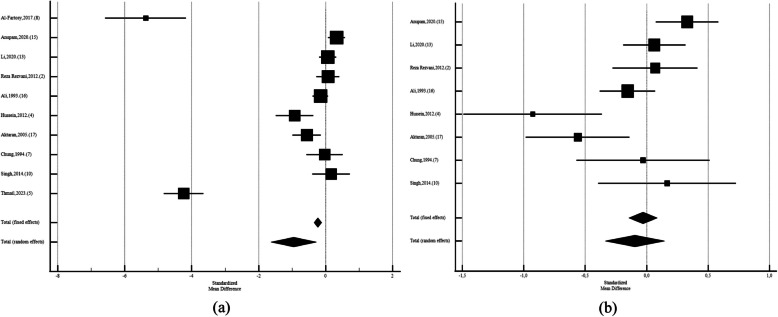
Forest plots of meta-analysis of the levels of Follicle-Stimulating Hormone among the Asian diabetic and non-diabetic ones. Data are pooled SMDs with 95% Cis. **a** before leave out Al-Fartosy, Thmail,2023. (5) **b**)after leave out Al-Fartosy,2017, Thmail,2023. (5)

**Fig. 5:**
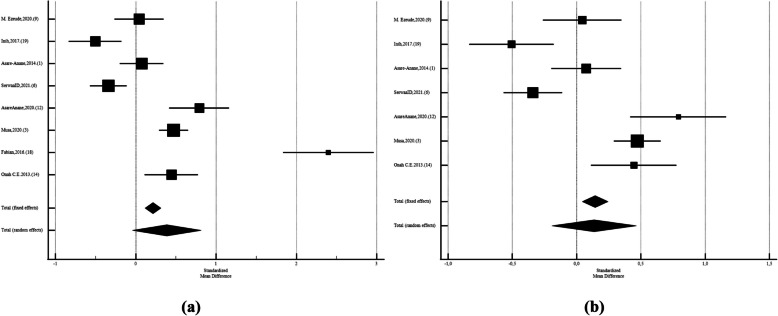
Forest plots of meta-analysis of the levels of Follicle-Stimulating Hormone among the African diabetic and non-diabetic ones. Data are pooled SMDs with 95% CIs. **a** before and **b**) after leave out Fabian,2016. (18)

**Fig. 6:**
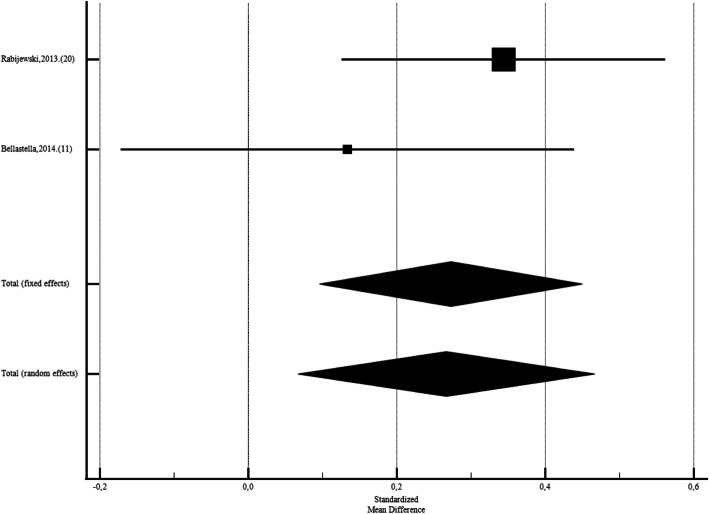
Forest plots of meta-analysis of the levels of Follicle-Stimulating Hormone among the European diabetic and non-diabetic ones. Data are pooled SMDs with 95% CI

Among Asian studies SDM was −0,955 (CI95%: −1,630 to −0,279; *p* = 0.006; I2: 96.91%; Egger's test: 0.03; Begg's test: 0.01, with diabetic men had lower FSH than control group. After leaving out studies that performed by Thmail, et al. (2023) [9], Al-Fartosy,et al. (2017) [39] (SMD: −0,0957;CI95%:−0,337 to 0,145;*P* = 0.43;I2: 73.67%; Egger's test:0.29; Begg's test:0.32), there were no significant diffrences (Fig. 4). While Among African diabetic males the FSH levels was not different than non-diabetics (SMD: 0,386; CI95%: −0,0401 to 0,813; *p* = 0.07; I2: 94.26%; Egger's test: 0.31; Begg's test: 0.21). After leave out Fabian,2016. (18) SMD: 0,136; CI95%: −0,192 to 0,464; *p* = 0.41; I2: 90.15%; Egger's test: 0.84; Begg's test: 0.65) also, it was not siginificant (Figs. 5). Also, among European men the FSH levels was significantly different than non-diabetics (SMD: 0,273; CI95%:0,0960 to 0,450; *p* = 0.003; I2: 18.41%; Egger's test: *P* < 0,0001; Begg's test: 0.31) (Figs. 6).

## Discussion

The current meta-analysis, which included data from 20 independent investigations, provides robust evidence that serum FSH levels are lower in men diagnosed with T2DM compared to non-diabetic control individuals. When stratified by continent, our analysis revealed that Asian men with diabetic men exhibited lower FSH levels compared to non-diabetic control. In contrast, no significant differences in FSH levels were observed among African diabetic males compared to non-diabetics. Also, among European men (2 studies) the FSH levels was significantly different than non-diabetics.

Men across the midlife are predisposed to an increased risk of developing T2DM, a susceptibility influenced by a combination of biological and environmental factors [49]. Recent studies have highlighted the intricate crosstalk between gonadotropins and metabolic regulation, with FSH signaling pathways implicated in the modulation of pancreatic β-cell function and insulin sensitivity [50]. However, the precise relationship between altered FSH levels and the presence of T2DM remains incompletely understood, necessitating further investigation for a definitive explanation. Clinical investigations have reported a higher prevalence of hypogonadism and altered gonadotropin levels in men with T2DM; approximately one-quarter of men diagnosed with T2DM exhibit aberrant levels of gonadotropins and testosterone, suggesting a potential interplay between hormonal imbalances and diabetes pathophysiology [51, 52].

Gonadotropin-releasing hormone (GnRH) can also cause small alteration in parameters of carbohydrate metabolism (35). In line with this, a study showed that using GnRH among men with prostate cancer resulted in worse glycemic status (36). On the other hand, low levels of sex hormone binding globulin (SHBG) and testosterone may play a role in the pathogenesis of T2DM. The results of a study on 225 subjects showed that SHBG levels were negatively associated with fasting glycemia, but no significant correlation was observed between SHBG and fasting insulin levels (37). This association is thought to be mediated, at least in part, by the influence of glucose on SHBG synthesis in the liver. These findings are consistent with previous prospective studies reporting that higher baseline SHBG predicts a lower incidence of T2DM, potentially due to the inhibitory effects of SHBG on the bioavailability of sex steroids, which have been linked to glucose dysregulation, which supports the central role of glucose in the regulation of SHBG. In addition SHBG can also affect liver glycogenesis, which is the most important factor in fasting blood sugar regulation [53]. Complex interaction between diabetes and hypothalamus-pituitary–testicular axis can decrease acute releasable pool of pituitary gonadotropins and following that leads to the reduced LH and FSH response [14]. What is important that the medication in diabetic men can affect the gonadotropins concentration? A pilot study showed that among hypogonadal men the changes in fasting blood glucose and HOMA1-IR were more pronounced in men with normal testosterone levels and using metformin reduced the FSH and LH levels [54]. It is also proposed that male reproductive dysfunction can be induced by hyperglycemia [55].

Furthermore, there is a close link between metabolic disturbances and reproductive dysfunctions [56]. A study reported that among males with unexplained infertility the higher levels of FSH and insulin was observed [57]. Increased insulin resistance and hyperglycemia has been noted as triggering factors of infertility in men [58]. According to studies, there is a bidirectional association between hypogonadism, T2DM and metabolic syndrome. It is unclear which of them comes first [59]. It is proposed that since young men with newly diagnosed DM suffer from hypogonadism, it might act as a precursor of T2DM [51]. Results from a cross-sectional study reported that almost 15% of men with primary couple’s infertility suffered from glycemic impairment [60]. Diabetic and obese men due to the metabolic changes, inflammatory disturbance and hormonal dysfunction are at risk of subfertility [61]. Earlier systematic review supported the role of physical activity in the improvement of FSH concentration of diabetic men [62]. More than half of diabetic men experience sexual dysfunction [63]. Erectile dysfunction is approximately 3.5 times more common in men with diabetes [64]. Endocrine imbalance and abnormalities in secretion of LH and FSH is considered one of potential diabetes-induced male infertility and sexual dysfunction mechanisms [65]. Diabetes by collection of pre-testicular effect ( decrease FSH and LH level and leydig cell function), testicular effect (increases ROS, decreasing antioxidant enzymes, abnormal cell apoptosis) and post-testicular effect (Erectile dysfunction and abnormal sexual behavior) can result in abnormalities in sex hormones and sexual dysfunction [66].

Obesity represents a plausible intermediary factor linking gonadotropin levels to the pathogenesis of diabetes. Numerous studies have elucidated the intricate interplay between gonadotropins, obesity, and diabetes. It has been shown that obese men suffered from decreased levels of gonadotropins (FSH and LH) and inhibin B follicle [67, 68]. Due to the existence of aromatase activity in fat tissue, it is possible that in obese men, testosterone is converted into estradiol and this estradiol suppresses the secretion of LH and FSH [69]. The metabolic disorders are associated with several complications including hypogonadism which is related to adiposity and insulin resistance [70].

Our finding showed that diabetic men had lower levels of FSH compared to the healthy men. Among included studies, there are inconsistencies in results, which affect the pooled SMD. On the one hand, some studies found higher levels of FSH, some others reported lower levels of FSH in diabetic men; on the other hand, some of them found non-significant differences between diabetic and those without diabetes. Variation in characteristics of the population of studies in terms of biological and environmental factors might explain these inconsistencies. The results of a study showed that younger people aged 18 to 35 years with type 2 diabetes have lower levels of testosterone, LH and FSH and higher prevalence of hypogonadotropic hypogonadism compared to type 1 diabetes [71]. However, some studies reported that androgens might be a better indicator for the incidence of diabetes than gonadal hormones [44, 46]. A study not only failed to support the association of FSH and diabetes, but also the non-significant association was observed between FSH and HbA1c [44]. By contrast, two old evidence revealed that diabetic men had significantly low serum LH and FSH [7, 8]. There is also age-related alteration in circulation of FSH and when a man ages, a significant increase in FSH levels is expected [72]. So, this alteration might change the risk of metabolic disturbances. A recent study revealed that older age and higher FSH values can predict the prediabetes status among men [60].

In this study, while our findings suggest diabetes may have a more pronounced suppressive effect on FSH in Asian men, but not African men, the reasons are not fully clear. Research going beyond documenting differences to explaining them is scarce. It may be assumed that diabetes has a more pronounced suppressive effect on FSH secretion in Asian men compared to African men due to genetic, epigenetic or environmental factors that influence the HPG axis response to the diabetic state in an ethnic-specific manner. This assumption needs to be investigated in a well-designed comprehensive study including different races. The other possible explanation is that, there is racial differences and disparities in development and incidence of T2DM, differences in physical environment, health care, and social context can contribute into this important [73]. There may also be due to the differences in the severity or duration of diabetes; if Asian diabetic men in the studies had more severe or longer-standing diabetes compared to African men, this could lead to greater disruption of FSH regulation. Factors like age, body composition, lifestyle, and comorbidities were not well-matched between the ethnic groups. Differences in these variables could influence FSH independent of diabetes status and mask or exaggerate ethnic differences; these factors can affect the regulation of releasing gonadotropins like inflammation, stress, drugs, metabolism and sex-steroids [74].

### Strengths and limitations

This study had some limitations and strengths that should be kept in mind. The search for databases was limited to the English language. Also, the studies mainly limited to Asia and Africa and there was lack of evidence from all other continents and the existent studies limited to a small sample size; so, the results cannot generalize to all men. The included studies were observational, unable us to explain possible cause–effect relationships. All studies rely on single FSH measurements. And, there might be variation in laboratory assessment of FSH in different studies. The majority of included studies are not population-based studies, future studies need to have long-term population-based design to investigate the association of FSH and risk of T2DM among men. Regarding the strength of this study, this is the first meta-analysis that comprehensively estimates the difference in FSH levels in men with T2DM compared to those without it.

## Conclusion

Our meta-analysis indicated that FSH concentration among diabetic men was lower than those without it. However, this difference was not observed among African men. This meta-analysis recommended future studies for assessment of the utility of FSH for risk assessment of T2DM among males.

## Supplementary Information

Supplementary Material 1.

## Data Availability

Some or all dataset generated during the current study are not publicly available but are available from the corresponding author on reasonable request.

## Abbreviations

FSH: Follicle-Stimulating Hormone
T2DM: Type 2 diabetes mellitus
CI: Confidence intervals
SMD: Standardized mean difference
NOS: Newcastle–Ottawa Quality Assessment Scale

## References

[1] Khan MAB, Hashim MJ, King JK, et al. Epidemiology of Type 2 Diabetes - Global Burden of Disease and Forecasted Trends. J Epidemiol Glob Health. 2020;10(1):107–11. 10.2991/jegh.k.191028.001. 32175717 10.2991/jegh.k.191028.001 PMC7310804

[2] Forouhi NG, Wareham NJ. Epidemiology of diabetes. Medicine. 2022;50(10):638–43. 10.1016/j.mpmed.2014.09.007. 10.1016/j.mpmed.2014.09.007 PMC4282306 25568613

[3] Kautzky-Willer A, Leutner M. Sex differences in type 2 diabetes. 2023;66(6):986-1002. 10.1007/s00125-023-05891-x 10.1007/s00125-023-05891-x PMC10163139 36897358

[4] Dilixiati D, Waili A, Tuerxunmaimaiti A, et al. Risk factors for erectile dysfunction in diabetes mellitus: a systematic review and meta-analysis. Front Endocrinol. 2024;15:1368079. 10.3389/fendo.2024.1368079. 10.3389/fendo.2024.1368079 PMC11024441 38638136

[5] Hussein WN, Mohammed ZM, Mohammed AN. Identifying risk factors associated with type 2 diabetes based on data analysis. Measurement: Sensors. 2022;24:100543. 10.1016/j.measen.2022.100543

[6] Ding EL, Song Y, Malik VS, Liu S. Sex differences of endogenous sex hormones and risk of type 2 diabetes: a systematic review and meta-analysis. JAMA. 2006;295(11):1288–99. 10.1001/jama.295.11.1288. 16537739 10.1001/jama.295.11.1288

[7] Maneesh M, Jayalakshmi H, Singh T, Chakrabarti A. Impaired hypothalamic-pituitary-gonadal axis function in men with diabetes mellitus. Indian J Clin Biochem. 2006;21:165–8. 10.1007/BF02913088. 23105591 10.1007/BF02913088 PMC3453761

[8] Hussein Z, Al-Qaisi J. Effect of diabetes mellitus type 2 on pituitary gland hormones (FSH, LH) in men and women in Iraq. Al-Nahrain Journal of Science. 2012;15(3):75–9. 10.22401/JNUS.15.3.11.

[9] Thmail BA, Hussain MM, Farhan AR. Association between gonadotrophic hormones (FSH and LH) and Type 2 diabetes mellitus in Adult Iraqi Males: a case-control Study. Adv Life Sci. 2023;10:25–9.

[10] Serwaa D, Bello FA, Osungbade KO, et al. Prevalence and determinants of low testosterone levels in men with type 2 diabetes mellitus; a case-control study in a district hospital in Ghana. Plos Glob Public Health. 2021;1(12):e0000052. 10.1371/journal.pgph.0000052. 36962255 10.1371/journal.pgph.0000052 PMC10021198

[11] Musa E, El-Bashir JM, Sani-Bello F, Bakari AG. Hypergonadotropic hypogonadism in Nigerian men with type 2 diabetes mellitus. Clinical Diabetology. 2021;10(1):129–37.

[12] Asare-Anane H, Ofori E, Agyemang Y, et al. Obesity and testosterone levels in Ghanaian men with type 2 diabetes. Clin Diab. 2014;32(2):61–5. 10.2337/diaclin.32.2.61. 10.2337/diaclin.32.2.61 PMC4485250 26130863

[13] Rezvani MR, Saadatjoo SA, Sorouri S, Fard MH. Comparison of serum free testosterone, luteinizing hormone and follicle stimulating hormone levels in diabetics and non-diabetics men-a case-control study. Journal of Research in Health Sciences. 2012;12(2). PMID: 23241519. 23241519

[14] Baccetti B, La Marca A, Piomboni P, et al. Insulin-dependent diabetes in men is associated with hypothalamo-pituitary derangement and with impairment in semen quality. Hum Reprod. 2002;17(10):2673–7. 10.1093/humrep/17.10.2673. 12351547 10.1093/humrep/17.10.2673

[15] Spaziani M, Carlomagno F. Extra-gonadal and non-canonical effects of FSH in males. Pharmaceuticals (Basel). 2023;16(6):813. 10.3390/ph16060813. (PMID: 37375761). 37375761 10.3390/ph16060813 PMC10300833

[16] Gordetsky J, van Wijngaarden E, O’Brien J. Redefining abnormal follicle-stimulating hormone in the male infertility population. BJU Int. 2012;110(4):568–72. 10.1111/j.1464-410X.2011.10783.x. 22177092 10.1111/j.1464-410X.2011.10783.x

[17] Fantus RJ, Lin JS, Chang C, et al. Compensated hypospermatogenesis: elevated follicle-stimulating hormone predicts decline in semen parameters among men with normal index semen analysis. Urology. 2023;174:99–103. 10.1016/j.urology.2023.01.015. 36716824 10.1016/j.urology.2023.01.015

[18] Grande G, Graziani A, Scafa R, et al. FSH Therapy in Male Factor Infertility: Evidence and Factors Which Might Predict the Response. Life. 2024;14(8):969. 10.3390/life14080969. 39202711 10.3390/life14080969 PMC11355377

[19] Caroppo E, Niederberger CS. Follicle-stimulating hormone treatment for male factor infertility. Fertil Steril. 2023;119(2):173–9. 10.1016/j.fertnstert.2022.09.362. 36470702 10.1016/j.fertnstert.2022.09.362

[20] Poljak Z, Hulin I, Maruscakova L, Mladosievicova B. Are GnRH and FSH potentially damaging factors in the cardiovascular system? Pharmazie. 2018;73(4):187–90. 10.1691/ph.2018.7992. 29609683 10.1691/ph.2018.7992

[21] Spaziani M, Carlomagno F, Tenuta M, et al. Extra-gonadal and non-canonical effects of FSH in Males. Pharmaceuticals. 2023;16(6):813. 10.3390/ph16060813. 37375761 10.3390/ph16060813 PMC10300833

[22] Shamseer L, Moher D, Clarke M, et al. Preferred reporting items for systematic review and meta-analysis protocols (PRISMA-P) 2015: elaboration and explanation. BMJ. 2015;349.10.1136/bmj.g7647 10.1136/bmj.g7647 25555855

[23] Stang A. Critical evaluation of the Newcastle-Ottawa scale for the assessment of the quality of nonrandomized studies in meta-analyses. Eur J Epidemiol. 2010;25:603–5. 10.1007/s10654-010-9491-z. 20652370 10.1007/s10654-010-9491-z

[24] Wells GA, Shea B, O’Connell D, et al. The Newcastle-Ottawa Scale (NOS) for assessing the quality of nonrandomised studies in meta-analyses. 2000.

[25] Achemlal L, Tellal S, Rkiouak F, et al. Bone metabolism in male patients with type 2 diabetes. Clin Rheumatol. 2005;24:493–6. 10.1007/s10067-004-1070-9. 15747054 10.1007/s10067-004-1070-9

[26] Cao J, Li J, Hao W, et al. Correlation of sex hormone and androgen receptor with diabetes mellitus in elderly men. Aging Male. 2011;14(3):162–7. 10.3109/13685538.2011.575479. 21574908 10.3109/13685538.2011.575479

[27] Handelsman D, Conway A, Boylan L, et al. Testicular function and glycemic control in diabetic men a controlled study. Andrologia. 1985;17(5):488–96. 10.1111/j.1439-0272.1985.tb01047.x. 3933383 10.1111/j.1439-0272.1985.tb01047.x

[28] Ficher M, Zuckerman M, Fishkin Re, et al. Do endocrines play an etiological role in diabetic and nondiabetic sexual dysfunctions? J Androl. 1984;5(1):8-16. 10.1002/j.1939-4640.1984.tb00771.x 10.1002/j.1939-4640.1984.tb00771.x 6423596

[29] Zeidler A, Gelfand R, Tamagna E, et al. Pituitary gonadal function in diabetic male patients with and without impotence. Andrologia. 1982;14(1):62–7. 10.1111/j.1439-0272.1982.tb03096.x. 6802026 10.1111/j.1439-0272.1982.tb03096.x

[30] Persky V, Abasilim C, Tsintsifas K, et al. Sex Hormones and Diabetes in 45- to 74-year-old men and postmenopausal women: The hispanic community health study. J Clin Endocrinol Metab. 2023;108(7):1709–26. 10.1210/clinem/dgad018. 36633580 10.1210/clinem/dgad018 PMC10271226

[31] Peng J, Li D, Liu L, et al. Comparison of characteristics between Chinese diabetes mellitus-induced erectile dysfunction populations and non-diabetes mellitus-induced erectile dysfunction populations: A cross-sectional study. Front Endocrinol. 2022;13:1096045. 10.3389/fendo.2022.1096045. 10.3389/fendo.2022.1096045 PMC9811585 36619568

[32] Gray A, Feldman Ha, Mckinlay JB, Longcope C. Age, disease, and changing sex hormone levels in middle-aged men: results of the Massachusetts Male Aging Study. J Clin Endocrinol Metab. 1991;73(5):1016-1025. 10.1210/jcem-73-5-1016 10.1210/jcem-73-5-1016 1719016

[33] Hashim NA, Madhi R, Al-Ali ZA, et al. Study on assessment of reproductive hormones in male patients with type 1 and 2 diabetes mellitus. hormones. 2024;1:T2DM. 10.5455/jabet.2024.d27

[34] Chang TC, Tung CC, Hsiao YL. Hormonal changes in elderly men with non-insulin-dependent diabetes mellitus and the hormonal relationships to abdominal adiposity. Gerontology. 1994;40(5):260–7. 10.1159/000213594. 7959082 10.1159/000213594

[35] Anupam B, Shivaprasad C, Vijaya S, et al. Prevalence of hypogonadism in patients with type 2 diabetes mellitus among the Indian population. Diab Metab Syndrome. 2020;14(5):1299–304. 10.1016/j.dsx.2020.07.006 32755825

[36] Bellastella G, Maiorino MI, Olita L, et al. Vitamin D deficiency in type 2 diabetic patients with hypogonadism. J Sex Med. 2014;11(2):536–42. 10.1111/jsm.12384. 24238472 10.1111/jsm.12384

[37] Asare-Anane H, Ofori EK, Kwao-Zigah G, et al. Lower circulating kisspeptin and primary hypogonadism in men with type 2 diabetes. Endocrinol Diab Metab. 2019;2(3):e00070. 10.1002/edm2.70. 10.1002/edm2.70 PMC8565652 34505408

[38] Onah C, Meludu S, Dioka C, et al. Pattern of male sex hormones in type 2 diabetic patients in Nnewi South Eastern Nigeria. IOSR-JDMS. 2013;10(4):65–70. 10.9790/0853-1046570.

[39] Fabian UA, Charles-Davies MA, Fasanmade AA, et al. Male sexual dysfunction, leptin, pituitary and gonadal hormones in Nigerian males with metabolic syndrome and type 2 diabetes mellitus. J Reprod Infertil. 2016;17(1):17 (PMCID: PMC4769850). 26962479 PMC4769850

[40] Inih OS, Esther YE, Adetola FO, et al. Testicular dysfunction is a common feature in men with type 2 diabetes mellitus in a Nigerian tertiary hospital. Curr Diabetes Rev. 2018;14(3):298–306. 10.51253/pafmj.v74i6.9906. 28443501 10.2174/1573399813666170425152046

[41] Aktaran S, Akarsu E, Meram İ, et al. Correlation of increased lipid peroxidation with serum gonadotropins and testosterone levels in type 2 diabetic men with erectile dysfunction. Turkish J Endocrinol Metab. 2005;4:119–24.

[42] Al-Fartosy AJM, Mohammed IM. Biochemical study of the effects of insulin resistance on sex hormones in men and women type-2 diabetic patients/Meisan-Iraq. Adv Biochem. 2017;5(5):79–88.

[43] Singh AK, Tomarz S, Chaudhari AR, et al. Type 2 diabetes mellitus affects male fertility potential. Indian J Physiol Pharmacol. 2014;58(4):403–6 (PMID: 26215009). 26215009

[44] Rezvani MR, Saadatjou SA, Sorouri S, Fard MH. Comparison of serum free testosterone, luteinizing hormone and follicle stimulating hormone levels in diabetics and non-diabetics men-a case-control study. J Res Health Sci. 2012;12(2):98–100 (PMID: 23241519). 23241519

[45] Ezeude CM, Ezeude AM, Anyanwu AC, et al. Erectile dysfunction in a Cohort of Eugonodal type 2 diabetic men attending a tertiary healthcare facility: prevalence and correlation with testicular volume. J Endo and Dis. 2020;4(1):2640–1045.

[46] Li N, Huang C, Lan B, et al. Association of gonadal hormones and sex hormone binding globulin with risk of diabetes: A cohort study in middle-aged and elderly Chinese males. Int J Clin Pract. 2021;75(5):e14008. 10.1111/ijcp.14008. 33400357 10.1111/ijcp.14008

[47] Rabijewski M, Papierska L, Zgliczyński W, Piątkiewicz P. The incidence of hypogonadotropic hypogonadism in type 2 diabetic men in Polish population. Biomed Res Int. 2013;2013(1):767496. 10.1155/2013/767496. 24222915 10.1155/2013/767496 PMC3810490

[48] Ali ST, Shaikh RN, Ashfaqsiddiqi N, Siddiqi PQ. Serum and urinary levels of pituitary-gonadal hormones in insulin-dependent and non-insulin-dependent diabetic males with and without neuropathy. Arch Androl. 1993;30(2):117–23. 10.3109/01485019308987744. 8470941 10.3109/01485019308987744

[49] Huebschmann AG, Huxley RR, Kohrt WM, et al. Sex differences in the burden of type 2 diabetes and cardiovascular risk across the life course. Diabetologia. 2019;62:1761–72. 10.1007/s00125-019-4939-5. 31451872 10.1007/s00125-019-4939-5 PMC7008947

[50] Cheng Y, Zhu H, Ren J, et al. Follicle-stimulating hormone orchestrates glucose-stimulated insulin secretion of pancreatic islets. Nat Commun. 2023;14(1):6991. 10.1038/s41467-023-42801-6. 37914684 10.1038/s41467-023-42801-6 PMC10620214

[51] Dandona P, Dhindsa S. Update: Hypogonadotropic hypogonadism in type 2 diabetes and obesity. J Clin Endocrinol Metab. 2011;96(9):2643–51. 10.1210/jc.2010-2724. 21896895 10.1210/jc.2010-2724 PMC3167667

[52] Al Hayek AA, Robert AA, Alshammari G, et al. assessment of hypogonadism in men with type 2 diabetes: A cross-sectional study from Saudi Arabia. Clin Med Insights Endocrinol Diabetes. 2017;19(10):1179551417710209. 10.1177/1179551417710209. 10.1177/1179551417710209 PMC5439570 28579862

[53] Peter A, Kantartzis K, Machann J, et al. Relationships of circulating sex hormone–binding globulin with metabolic traits in humans. Diabetes. 2010;59(12):3167–73. 10.2337/db10-0179. 20841609 10.2337/db10-0179 PMC2992779

[54] Krysiak R, Szkróbka W, Bednarska-Czerwińska A, Okopień B. Plasma gonadotropin levels in metformin-treated men with prediabetes: a non-randomized, uncontrolled pilot study. Fundam Clin Pharmacol. 2021;35(2):466–72. 10.1111/fcp.12600. 32813271 10.1111/fcp.12600

[55] Maresch CC, Stute DC, Alves MG, et al. Diabetes-induced hyperglycemia impairs male reproductive function: a systematic review. Hum Reprod Update. 2017;24(1):86–105. 10.1093/humupd/dmx033. 10.1093/humupd/dmx033 29136166

[56] Morelli A, Comeglio P, Sarchielli E, et al. Negative effects of high glucose exposure in human gonadotropin-releasing hormone neurons. Int J Endocrinol. 2013;2013:684659. 10.1155/2013/684659. 24489542 10.1155/2013/684659 PMC3893744

[57] Mansour R, El-Faissal Y, Kamel A, et al. Increased insulin resistance in men with unexplained infertility. Reprod Biomed Online. 2017;35(5):571–5. 10.1016/j.rbmo.2017.08.020. 28888863 10.1016/j.rbmo.2017.08.020

[58] George BT, Jhancy M, Dube R, et al. The Molecular Basis of Male Infertility in Obesity: A Literature Review. Int J Mol Sci. 2023;25(1):179. 10.3390/ijms25010179. 38203349 10.3390/ijms25010179 PMC10779000

[59] Dandona P, Dhindsa S, Chaudhuri A, et al. Hypogonadotrophic hypogonadism in type 2 diabetes, obesity and the metabolic syndrome. Curr Mol Med. 2008;8(8):816–28. 10.2174/156652408786733658. 19075678 10.2174/156652408786733658

[60] Boeri L, Capogrosso P, Ventimiglia E, et al. Undiagnosed prediabetes is highly prevalent in primary infertile men–results from a cross-sectional study. BJU Int. 2019;123(6):1070–7. 10.1111/bju.14558. 30328251 10.1111/bju.14558

[61] AbbasiHormozi S, Kouhkan A, Shahverdi A, et al. How much obesity and diabetes do impair male fertility? Reprod Biol Endocrinol. 2023;21(1):48. 10.1186/s12958-022-01034-w. 37208686 10.1186/s12958-022-01034-w PMC10197395

[62] Lavín-Pérez AM, Collado-Mateo D, Villafaina S, Calle-Guisado V. The Role of Exercise to Reduce the Impact of Diabetes in the Seminal Quality: A Systematic Review. Medicina (Kaunas). 2021;57(2). 10.3390/medicina57020159 10.3390/medicina57020159 PMC7916555 33578871

[63] Gebeyehu NA, Gesese MM, Tegegne KD, et al. Global prevalence of sexual dysfunction among diabetic patients from 2008 to 2022: Systematic review and meta-analysis. Metabolism Open. 2023;18:100247. 10.1016/j.metop.2023.100247. 37323562 10.1016/j.metop.2023.100247 PMC10267599

[64] Kouidrat Y, Pizzol D, Cosco T, et al. High prevalence of erectile dysfunction in diabetes: a systematic review and meta-analysis of 145 studies. Diabet Med. 2017;34(9):1185–92. 10.1111/dme.13403. 28722225 10.1111/dme.13403

[65] Huang R, Chen J, Guo B, et al. Diabetes-induced male infertility: potential mechanisms and treatment options. Mol Med. 2024;30(1):11. 10.1186/s10020-023-00771-x. 38225568 10.1186/s10020-023-00771-x PMC10790413

[66] Andlib N, Sajad M, Kumar R, Thakur SC. Abnormalities in sex hormones and sexual dysfunction in males with diabetes mellitus: A mechanistic insight. Acta Histochem. 2023;125(1):151974. 10.1016/j.acthis.2022.151974. 36455338 10.1016/j.acthis.2022.151974

[67] Pauli EM, Legro RS, Demers LM, et al. Diminished paternity and gonadal function with increasing obesity in men. Fertil Steril. 2008;90(2):346–51. 10.1016/j.fertnstert.2007.06.046. 18291378 10.1016/j.fertnstert.2007.06.046 PMC2597471

[68] Ekpor E, Akyirem S, Adade Duodu P. Prevalence and associated factors of overweight and obesity among persons with type 2 diabetes in Africa: a systematic review and meta-analysis. Ann Med. 2023;55(1):696–713. 10.1080/07853890.2023.2182909. 36821504 10.1080/07853890.2023.2182909 PMC9970251

[69] Stárka L, Hill M, Pospíšilová H, Dušková M. Estradiol, obesity and hypogonadism. Physiol Res. 2020;69(Suppl 2):S273–s278. 10.33549/physiolres.934510. 33094625 10.33549/physiolres.934510 PMC8603736

[70] Kapoor D, Aldred H, Clark S, et al. Clinical and biochemical assessment of hypogonadism in men with type 2 diabetes: correlations with bioavailable testosterone and visceral adiposity. Diabetes Care. 2007;30(4):911–7. 10.2337/dc06-1426. 17392552 10.2337/dc06-1426

[71] Chandel A, Dhindsa S, Topiwala S, et al. Testosterone concentration in young patients with diabetes. Diabetes Care. 2008;31(10):2013–7. 10.2337/dc08-0851. 18650372 10.2337/dc08-0851 PMC2551646

[72] Elmlinger MW, Dengler T, Weinstock C, Kuehnel W. Endocrine alterations in the aging male. Clin Chem Lab Med. 2003;41(7):934–41. 10.1515/CCLM.2003.142. (PMID: 12940521). 12940521 10.1515/CCLM.2003.142

[73] Hill-Briggs F, Adler NE, Berkowitz SA. Social determinants of health and diabetes: A scientific review. Diabetes Care. 2020;44(1):258–79. 10.2337/dci20-0053. 33139407 10.2337/dci20-0053 PMC7783927

[74] Marques P, Skorupskaite K, Rozario KS, et al. Physiology of GNRH and gonadotropin secretion. Endotext. 2022. South Dartmouth (MA): MDText.com, Inc.; 2000-. Available from: https://www.ncbi.nlm.nih.gov/books/NBK279070/

