# Supplementary material for: A systematic review and meta-analysis of follicle-stimulating hormone levels among men with type 2 diabetes
Source: Basic Clin Androl. 2025 Mar 14;35:11. doi: 10.1186/s12610-025-00257-2 (PMC11908071; doi:10.1186/s12610-025-00257-2)
Supplement: Supplementary file 1 — Supplementary Material 1. [file 12610_2025_257_MOESM1_ESM.docx]

**Supplementary file**

**Title: A Systematic Review and Meta-analysis of Follicle-Stimulating Hormone levels among Men with type 2 Diabetes**

**Name of authors**: Fahimeh Ramezani Tehrani^1^, Vida Ghasemi ^2^, Marzieh Saei Ghare Naz ^1*^

1. Reproductive Endocrinology Research Center, Research Institute for Endocrine Sciences, Shahid Beheshti University of Medical Sciences, Tehran, Iran.

2. Asadabad School of Medical Sciences, Asadabad, Iran.

Supplementary Table 1: Search strategy

| set | Search strategy PubMed |
| --- | --- |
| #1 | **((Follicle-Stimulating Hormone[Title/Abstract]) OR (Follitropin[Title/Abstract])) OR (FSH, Human[Title/Abstract])) OR (Human Follicle Stimulating Hormone[Title/Abstract])) OR (Human FSH[Title/Abstract])) OR (hFSH[Title/Abstract])) OR (Gonadotropins))** |
| #2 | **(((((((Diabetes Mellitus[Title/Abstract]) OR (Diabetes[Title/Abstract])) OR (glucose intolerance[Title/Abstract])) OR (Intolerance, Glucose[Title/Abstract])) OR (Impaired Glucose Tolerance[Title/Abstract])) OR (Tolerance, Impaired Glucose[Title/Abstract])) OR (Glucose Tolerances, Impaired[Title/Abstract]))** |
| #3 | #1 AND #2 |
| set | Search strategy WOS |
| #1 | Follicle-Stimulating Hormone (Topic) or Follitropin (Topic) or FSH, Human (Topic) or Human Follicle Stimulating Hormone (Topic) or Human FSH (Topic) or hFSH (Topic) or Gonadotropins (Topic) |
| #2 | Diabetes Mellitus (Topic) or Diabetes (Topic) or glucose intolerance (Topic) or Intolerance, Glucose (Topic) or Impaired Glucose Tolerance (Topic) or Tolerance, Impaired Glucose (Topic) or Glucose Tolerances, Impaired (Topic) |
| #3 | #1 AND #2 |
| set | Search strategy Epistemonikos |
|  | (title:((title:(Follicle-Stimulating Hormone) OR abstract:(Follicle-Stimulating Hormone)) OR (title:(Follitropin) OR abstract:(Follitropin)) OR (title:(FSH, Human) OR abstract:(FSH, Human)) OR (title:(Human Follicle Stimulating Hormone) OR abstract:(Human Follicle Stimulating Hormone)) OR (title:(Human FSH) OR abstract:(Human FSH)) OR (title:(hFSH) OR abstract:(hFSH)) OR (title:(Gonadotropins) OR abstract:(Gonadotropins))) OR abstract:((title:(Follicle-Stimulating Hormone) OR abstract:(Follicle-Stimulating Hormone)) OR (title:(Follitropin) OR abstract:(Follitropin)) OR (title:(FSH, Human) OR abstract:(FSH, Human)) OR (title:(Human Follicle Stimulating Hormone) OR abstract:(Human Follicle Stimulating Hormone)) OR (title:(Human FSH) OR abstract:(Human FSH)) OR (title:(hFSH) OR abstract:(hFSH)) OR (title:(Gonadotropins) OR abstract:(Gonadotropins)))) AND (title:((title:(Diabetes Mellitus) OR abstract:(Diabetes Mellitus)) OR (title:(Diabetes) OR abstract:(Diabetes)) OR (title:(glucose intolerance) OR abstract:(glucose intolerance)) OR (title:(Intolerance, Glucose) OR abstract:(Intolerance, Glucose)) OR (title:(Impaired Glucose Tolerance) OR abstract:(Impaired Glucose Tolerance)) OR (title:(Tolerance, Impaired Glucose) OR abstract:(Tolerance, Impaired Glucose)) OR (title:(Glucose Tolerances, Impaired) OR abstract:(Glucose Tolerances, Impaired))) OR abstract:((title:(Diabetes Mellitus) OR abstract:(Diabetes Mellitus)) OR (title:(Diabetes) OR abstract:(Diabetes)) OR (title:(glucose intolerance) OR abstract:(glucose intolerance)) OR (title:(Intolerance, Glucose) OR abstract:(Intolerance, Glucose)) OR (title:(Impaired Glucose Tolerance) OR abstract:(Impaired Glucose Tolerance)) OR (title:(Tolerance, Impaired Glucose) OR abstract:(Tolerance, Impaired Glucose)) OR (title:(Glucose Tolerances, Impaired) OR abstract:(Glucose Tolerances, Impaired)))) |
| set | Search strategy Scopus |
|  | ( TITLE-ABS-KEY ( follicle-stimulating AND hormone ) OR TITLE-ABS-KEY ( follitropin ) OR TITLE-ABS-KEY ( fsh, AND human ) OR TITLE-ABS-KEY ( human AND follicle AND stimulating AND hormon ) OR TITLE-ABS-KEY ( human AND fsh ) OR TITLE-ABS-KEY ( hfsh ) OR TITLE-ABS-KEY ( gonadotropins ) ) AND ( TITLE-ABS-KEY ( diabetes AND mellitus ) OR TITLE-ABS-KEY ( diabetes ) OR TITLE-ABS-KEY ( glucose AND intolerance ) OR TITLE-ABS-KEY ( intolerance, AND glucose ) OR TITLE-ABS-KEY ( impaired AND glucose AND tolerance ) OR TITLE-ABS-KEY ( tolerance, AND impaired AND glucose ) OR TITLE-ABS-KEY ( glucose AND tolerances, AND impaired ) ) AND ( LIMIT-TO ( LANGUAGE , "English" ) ) AND ( LIMIT-TO ( EXACTKEYWORD , "Human" ) OR LIMIT-TO ( EXACTKEYWORD , "Humans" ) ) AND ( LIMIT-TO ( DOCTYPE , "ar" ) OR LIMIT-TO ( DOCTYPE , "re" ) ) |
| set | Search strategy Cochrane Library |
| #1 | Follicle-Stimulating Hormone:ti,ab,kw OR Follitropin:ti,ab,kw OR FSH, Human:ti,ab,kw OR Human Follicle Stimulating Hormon:ti,ab,kw OR Human FSH:ti,ab,kw OR hFSH:ti,ab,kw OR Gonadotropins:ti,ab,kw(Word variations have been searched) |
| #2 | Diabetes Mellitus:ti,ab,kw OR Diabetes:ti,ab,kw OR glucose intolerance:ti,ab,kw OR Intolerance, Glucose:ti,ab,kw OR Impaired Glucose Tolerance:ti,ab,kw (Word variations have been searched) |
| #3 | #1 AND #2 |

| Supplementary Table 2: Characteristics of included studies | | | | | | | | | | | |
| --- | --- | --- | --- | --- | --- | --- | --- | --- | --- | --- | --- |
| **Authors, year**  **(ref)** | **Country** | **Sample size** | | **Mean±SD Age** | | **Follicle-Stimulating Hormone levels** | | | | | |
|  |  | **T2DM ^a^** | **Controls** | **T2DM** | **Controls** | **T2DM** | | | **Controls** | | |
|  |  |  |  |  |  | **Mean** | **SD** | **Unit** | **Mean** | **SD** | **Unit** |
| Asare-Anane,et al. (2014) [1] | Ghana | 105 | 105 | 48.90±9.40 | 42.80±5.60 | 5.80 | 3.00 | IU/L | 5.60 | 2.30 | IU/L |
| Rezvani,et al. (2012) [2] | Iran | 65 | 65 | 52.95±1.69 | 53.22±1.81 | 5.27 | 4.74 | PG/ML | 5.02 | 1.92 | PG/ML |
| Musa,et al. (2020) [3] | Nigeria | 358 | 179 | 46.34±5.66 | 44.09±12.39 | 8.50 | 8.17 | IU/L | 5.17 | 3.89 | IU/L |
| Hussein,et al. (2012) [4] | Iraq | 30 | 25 | 57.40±10.60 | 60.60±11.20 | 11.04 | 1.85 | MUI/ML | 13.03 | 2.40 | MUI/ML |
| Thmail,et al. (2023) [5] | Iraq | 100 | 50 | 44.48±8.06 | 42.85±1.22 | 3.41 | 1.25 | IU/L | 9.01 | 1.44 | IU/L |
| SerwaaID,et al. (2021) [6] | Ghana | 150 | 150 | 58.25±9.71 | 56.34±9.40 | 7.19 | 4.68 | MUI/ML | 8.85 | 5.05 | MUI/ML |
| Chung, et al. (1994) [7] | Taiwan | 20 | 40 | 67.25±5.50 | 66.75±5.50 | 41.00 | 33.45 | MUI/ML | 42.02 | 35.56 | MUI/ML |
| Al-Fartosy,et al. (2017) [8] | Iraq | 26 | 25 | 52.37±6.46 | 51.66±7.72 | 7.24 | 1.14 | µU/ml | 12.79 | 0.87 | µU/ml |
| M. Ezeude,et al. (2020) [9] | Nigeria | 124 | 62 | 58.29±10.02 | 56.48±10.58 | 10.65 | 1.07 | MUI/ML | 10.60 | 1.23 | MUI/ML |
| Singh,et al. (2020) [10] | India | 25 | 25 | 47.80±3.00 | 44.30±2.30 | 7.40 | 1.20 | MUI/ML | 7.10 | 2.20 | MUI/ML |
| Bellastella et al. (2014) [11] | Italy | 71 | 100 | 54.8 ± 10.5 | 53.4 ± 10.9 | 3.4 | 0.88 | IU/L | 3.2 | 1.8 | IU/L |
| AsareAnane et al. (2020) [12] | Ghana | 60 | 60 | 49.37 ± 10.86 | 48.35 ± 7.91 | 15.83 | 9.29 | mlU/mL | 10.39 | 2.65 | mlU/mL |
| Li et al. (2020) [13] | China | 66 | 607 | 59.7 ± 7.4 | 57.5 ± 7.0 | 10.64 | 2.18 | mIU/L | 10.50 | 2.22 | mIU/L |
| Onah C.E (2013) [14] | Nigeria | 125 | 50 | 51.6±8.6 | 49.18±11.46 | 6.3 | 3.8 | μIU/ml | 4.8 | 1.8 | μIU/ml |
| Anupam et al, (2020) [15] | India | 150 | 100 | 50.6± 9.4 | 50.3± 8.5 | 7.1 | 4.1 | mIU/mL | 5.9 | 2.8 | mIU/mL |
| Ali et al, (1993) [16] | Pakistan | 314 | 100 | 15 to 81 years old | 15 to 81 years old | 8.5 | 3.6 | mIU/ml | 9 | 1.5 | mIU/ml |
| Aktaran et al, (2005)[17] | Turkey | 52 | 40 | 50.82±4.97 | 42.7±8.5 | 6.31 | 3.03 | mIU/ml | 7.7 | 1.4 | mIU/ml |
| Fabian et al, (2016)[18] | Nigeria | 34 | 53 | 21-90 years | 21-90 years | 16.5 | 2.5 | IU/l | 12 | 1.3 | IU/l |
| Inih et al (2017)[19] | Nigeria | 108 | 56 | 51.7 ± 5.9 | 50.9 ±4.6 | 6.19 | 2.17 | mIu/ml | 7.36 | 2.55 | mIu/ml |
| Rabijewski, et al (2013) [20] | Poland | 184 | 149 | 58.5 ± 2.3 | 59.6 ± 3.2 | 8.8 | 0.3 | mIU/L | 8.6 | 0.8 | mIU/L |

Abbreviation:

a: Type 2 diabetes

Supplementary figure 1. Quality assessment of included studies

**References:**

[1] Asare-Anane H, Ofori E, Agyemang Y, et al. Obesity and testosterone levels in Ghanaian men with type 2 diabetes. Clinical Diabetes. 2014;32(2):61-65.

[2] Rezvani MR, Saadatjou SA, Sorouri S, Fard MH. Comparison of serum free testosterone, luteinizing hormone and follicle stimulating hormone levels in diabetics and non-diabetics men-a case-control study. 2012.

[3] Musa E, El-Bashir JM, Sani-Bello F, Bakari AG. Hypergonadotropic hypogonadism in Nigerian men with type 2 diabetes mellitus. Clinical Diabetology. 2021;10(1):129-137.

[4] Hussein Z, Al-Qaisi J. Effect of diabetes mellitus type 2 on pituitary gland hormones (FSH, LH) in men and women in Iraq. Al-Nahrain Journal of Science. 2012;15(3):75-79.

[5] Thmail BA, Hussain MM, Farhan AR. Association between gonadotrophic hormones (FSH and LH) and Type 2 diabetes mellitus in Adult Iraqi Males: a case-control Study. Advancements in Life Sciences. 2023;10:25-29.

[6] Serwaa D, Bello FA, Osungbade KO, et al. Prevalence and determinants of low testosterone levels in men with type 2 diabetes mellitus; a case-control study in a district hospital in Ghana. PLOS Global Public Health. 2021;1(12):e0000052.

[7] Chang TC, Tung CC, Hsiao YL. Hormonal changes in elderly men with non-insulin-dependent diabetes mellitus and the hormonal relationships to abdominal adiposity [Article]. Gerontology. 1994;40(5):260-267.

[8] Al-Fartosy AJM, Mohammed IM. Biochemical study of the effects of insulin resistance on sex hormones in men and women type-2 diabetic patients/Meisan-Iraq. Advances in Biochemistry. 2017;5(5):79-88.

[9] Ezeude CM, Ezeude AM, Anyanwu AC, et al. Erectile Dysfunction in a Cohort of Eugonodal Type 2 Diabetic Men Attending a Tertiary Healthcare Facility: Prevalence and Correlation with Testicular Volume. J Endo and Dis. 2020;4(1):2640-1045.

[10] Singh AK, Tomarz S, Chaudhari AR, et al. Type 2 diabetes mellitus affects male fertility potential. Indian J Physiol Pharmacol. 2014;58(4):403-6.

[11] Bellastella G, Maiorino MI, Olita L, et al. Vitamin D deficiency in type 2 diabetic patients with hypogonadism. The Journal of Sexual Medicine. 2014;11(2):536-542.

[12] Asare‐Anane H, Ofori EK, Kwao‐Zigah G, et al. Lower circulating kisspeptin and primary hypogonadism in men with type 2 diabetes. Endocrinology, Diabetes & Metabolism. 2019;2(3):e00070.

[13] Li N, Huang C, Lan B, et al. Association of gonadal hormones and sex hormone binding globulin with risk of diabetes: A cohort study in middle‐aged and elderly Chinese males. International Journal of Clinical Practice. 2021;75(5):e14008.

[14] Onah C, Meludu S, Dioka C, et al. Pattern of male sex hormones in type 2 diabetic patients in Nnewi, South Eastern Nigeria. IOSR-JDMS. 2013;10(4):65-70.

[15] Anupam B, Shivaprasad C, Vijaya S, et al. Prevalence of hypogonadism in patients with type 2 diabetes mellitus among the Indian population. Diabetes & metabolic syndrome. 2020 Sep-Oct;14(5):1299-1304.

[16] Ali ST, Shaikh RN, Ashfaqsiddiqi N, Siddiqi PQ. Serum and urinary levels of pituitary--gonadal hormones in insulin-dependent and non-insulin-dependent diabetic males with and without neuropathy. Archives of andrology. 1993 Mar-Apr;30(2):117-23.

[17] Aktaran S, Akarsu E, Meram İ, et al. Correlation of increased lipid peroxidation with serum gonadotropins and testosterone levels in type 2 diabetic men with erectile dysfunction. Turkish J Endocrinol Metab. 2005;4:119-124.

[18] Fabian UA, Charles-Davies MA, Fasanmade AA, et al. Male sexual dysfunction, leptin, pituitary and gonadal hormones in Nigerian males with metabolic syndrome and type 2 diabetes mellitus. Journal of Reproduction & Infertility. 2016;17(1):17.

[19] Inih OS, Esther YE, Adetola FO, et al. Testicular dysfunction is a common feature in men with type 2 diabetes mellitus in a Nigerian tertiary hospital. Current diabetes reviews. 2018;14(3):298-306.

[20] Rabijewski M, Papierska L, Zgliczyński W, Piątkiewicz P. The incidence of hypogonadotropic hypogonadism in type 2 diabetic men in Polish population. BioMed research international. 2013;2013(1):767496.
